# Challenges and approaches in assessing the interplay between microorganisms and their physical micro-environments

**DOI:** 10.1016/j.csbj.2020.09.030

**Published:** 2020-10-01

**Authors:** Harry J. Harvey, Ricky D. Wildman, Sacha J. Mooney, Simon V. Avery

**Affiliations:** a School of Life Sciences, University of Nottingham, Nottingham, UK; b Faculty of Engineering, University of Nottingham, Nottingham, UK; c School of Biosciences, University of Nottingham, Nottingham, UK

**Keywords:** Microbial ecology, Structured environments, Microenvironment, Soil structure, Methodology, Micromodels

## Abstract

Spatial structure over scales ranging from nanometres to centimetres (and beyond) varies markedly in diverse habitats and the industry-relevant settings that support microbial activity. Developing an understanding of the interplay between a structured environment and the associated microbial processes and ecology is fundamental, but challenging. Several novel approaches have recently been developed and implemented to help address key questions for the field: from the use of imaging tools such as X-ray Computed Tomography to explore microbial growth in soils, to the fabrication of scratched materials to examine microbial-surface interactions, to the design of microfluidic devices to track microbial biofilm formation and the metabolic processes therein. This review discusses new approaches and challenges for incorporating structured elements into the study of microbial processes across different scales. We highlight how such methods can be pivotal for furthering our understanding of microbial interactions with their environments.

## 1 Introduction

### 1.1 Overview of structured environments and their interplay with microorganisms

**Fig. 1:**
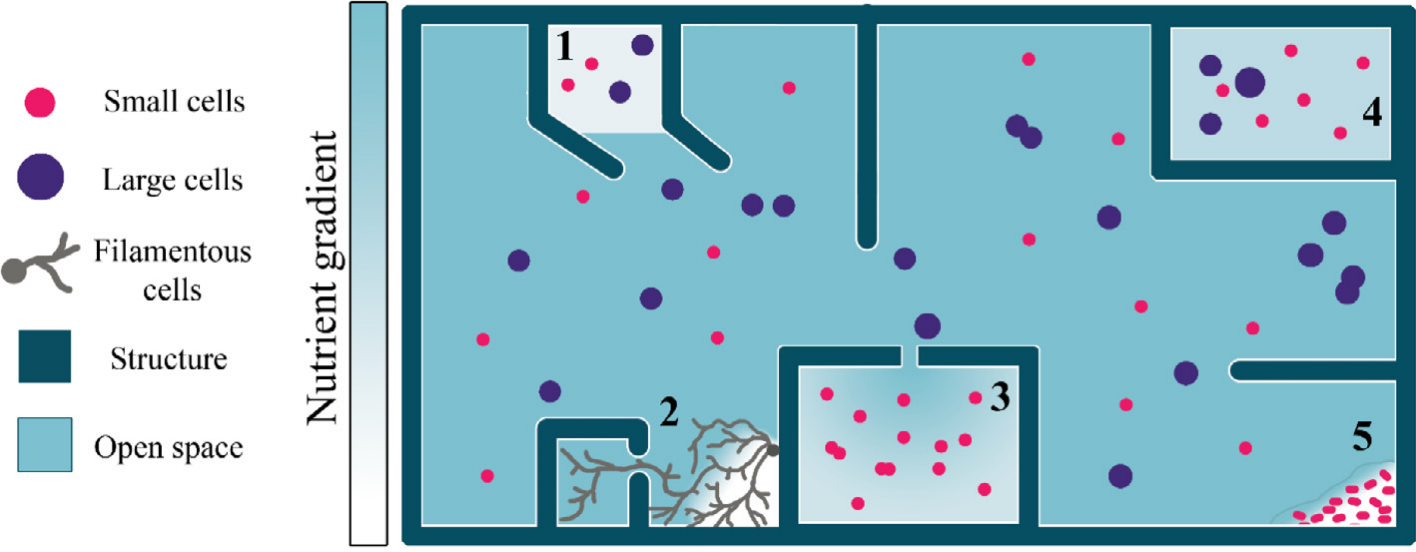
Simplified schematic of structured (micro)environments, as they may be modelled in the laboratory, and emergence of heterogeneous landscapes. Environments are typically structured by the division of space by physical structure, creating gradients of resources needed for growth, and the physical separation of microorganisms. Physical structures can create phase boundaries (1) which limit nutrient diffusion, as well as size-selective pores (3) and the separation of space into isolated environments (4) which can isolate both organisms and nutrients, potentially leading to localised nutrient depletion and localised adaptations by the organisms. Microorganisms can also create structure within their environment, such as by filamentous exploration (2) and biofilm formation (5) which can also limit local nutrient availability.

Almost ubiquitously, the environments of microorganisms have three-dimensional structure and create heterogeneous distributions of the space in which microorganisms reside. In this review, we refer to structured environments as habitats where the arrangement of the solid phase (pores/surfaces/walls) impose or facilitate the formation of spatial gradients and environmental heterogeneity (illustrated in Fig. 1). Broadly speaking, structured environments, from the nanometre to centimetre scale, produce a spatially heterogeneous distribution of abiotic factors such as nutrients, water, and oxygen [44], [4], [49] and of the microorganisms living within these environments. The extent of this heterogeneity also varies between environments. For example, nutrient concentrations may be more homogeneous when distributed in an entirely aqueous environment than one composed of both aqueous and solid or gaseous phases.

This organisation of abiotic and biotic factors can be determined by structure in several ways, such as the existence of spaces or pores of small sizes that exclude some organisms, or of oxygen gradients that are preferential to either anaerobic or aerobic organisms [4] (Fig. 1). This means that an environment structured in this way naturally establishes a heterogeneous landscape in which microorganisms fall into a range of niches to which they may, over time, adapt further.

There are many real-world examples that illustrate the impact that structured environments have on microbial life. A prime example is the soil habitat, which supports much of the planet’s biogeochemical activity, e.g., carbon and nitrogen cycling [11]. This is possible partly because of the complex nature of the soils’ constituent parts and the interaction of these to form cohesive porous structures [28]. For instance, the connectivity of pores can influence species diversity by creating a greater number of isolated habitats when pore-connectivity is low, increasing species diversity, and *vice versa* [8]. The non-uniform distribution of resources mentioned above is very relevant to soils, and mathematical modelling suggests that exploration of fungal hyphae towards nutrient hotspots in turn increases the structural complexity of that spot, as hyphae create channels while they penetrate through the soil [49].

Other examples of structured environments include non-natural surfaces that are prone to biofilm formation, such as micro-scratches in surfaces used in hygienic and medical settings [47] and porous biomedical devices (e.g. catheters, voice prosthesis, and porous scaffolding used to mimic bone) [18]. These structures can facilitate the persistence of biofilm-forming organisms after physical perturbation, such as when mechanically cleaning a surface or when fluid flow generates shear forces [15]. Biofilms themselves can be considered highly structured environments encompassing the spatial division of different species, of labour within a monospecies biofilm, and of gradients in nutrients or secreted-metabolites from cells (with the metabolites from some cells serving as substrates for others) [13].

In this review, we highlight recent approaches and challenges for incorporating structured elements into the study of microbial processes at different scales. These approaches are currently applied in the study of diverse environments and in different disciplines. Their collation here reflects the interdisciplinary approach that is increasingly required to capitalise on emerging technologies in order to enable a fuller understanding of the interplay between microorganisms and their structured environments.

## 2 Recent methods for investigating microbial (micro)environments

Considering the range of microbial habitats that have structure, it is very desirable to be able to study organisms either in their native structured environments or models thereof. The former typically poses considerable experimental challenges, many of which centre on two main concerns: (i) the difficulties of studying these environments under controlled conditions and with appropriate controls, and (ii) examining (pre-existing) environments non-destructively. In recent years, a range of new technical approaches and methodologies have been developed which help to address these challenges, among others, enabling new understanding of important biological processes as they occur in real environments.

### 2.1 X-ray Computed Tomography for imaging structured environments

X-ray Computed Tomography (CT) is an imaging technique in which incident beams of X-rays pass through a subject as it is rotated through 360°, and subsequently collected by CCD detectors. The beam is attenuated in a manner dependent on the X-ray absorption density of the subject being examined. By computational analysis of attenuation through all angles of the subject, horizontal and vertical cross sections through the subject can be generated to form a three-dimensional model. A comprehensive overview of the principles of X-ray CT is available in Maire and Withers [33]. After these images are digitally reconstructed, they can be quantitatively analysed to determine properties about the subject. Such properties can include pore volume, degree of anisotropy (the degree to which structures within the subject are oriented) and structure (or pore) connectivity.

X-ray CT was originally used in medical diagnosis, for example in detecting tissue death, tumours, and bone damage in humans and animals. However, this method has since become a versatile tool for the non-destructive investigation of many other types of structure [33] and is becoming more widely applied in the material, agricultural, and biological sciences [46], [22], [45]. Recently, X-ray CT has been used to study the (often reciprocal) interaction between biology and environment [38], discussed in further detail below.

#### 2.1.1 Microbial colonisation and movement in structured environments

Several studies have utilised X-ray CT for examining the role microorganisms play in modifying their habitat structure. One exemplar is Helliwell et al. [21] which used soil macrocosms produced from different soil types. They studied the impact of soil moisture and carbon input on microbial respiration and how the respiratory gas release relationship altered the soil porosity, pore shape and pore connectivity in the macrocosms. By comparing sterile and non-sterile macrocosms via repeated scanning over several weeks, it was demonstrated that the natural soil microbiota could significantly alter structure of its environment by hyphal exploration and gas release. This was especially marked following the addition of glucose, which elevated the rate of microbial respiration. It is also possible to gain some insight into soil structure by dissecting and analysing cross/thin sections of soil [7]. Thin sections are typically obtained by impregnating a soil with synthetic resin and then cutting with a diamond-tipped blade following a curing period prior to polishing. Polished sections can be mounted onto a microscope slide and cut or ground to achieve a desired thickness (typically around 30 µm) for examination and analysis. However, this approach is time consuming, destructive, and not three dimensional (unless many thin sections are re-constructed and assessed stereologically). Hence, X-ray CT helps to overcome such drawbacks, derives a significantly larger data set (c. 2000 images per sample) and facilitates new understanding via exploration of a soil's 3D spatial arrangement.

**Fig. 2:**
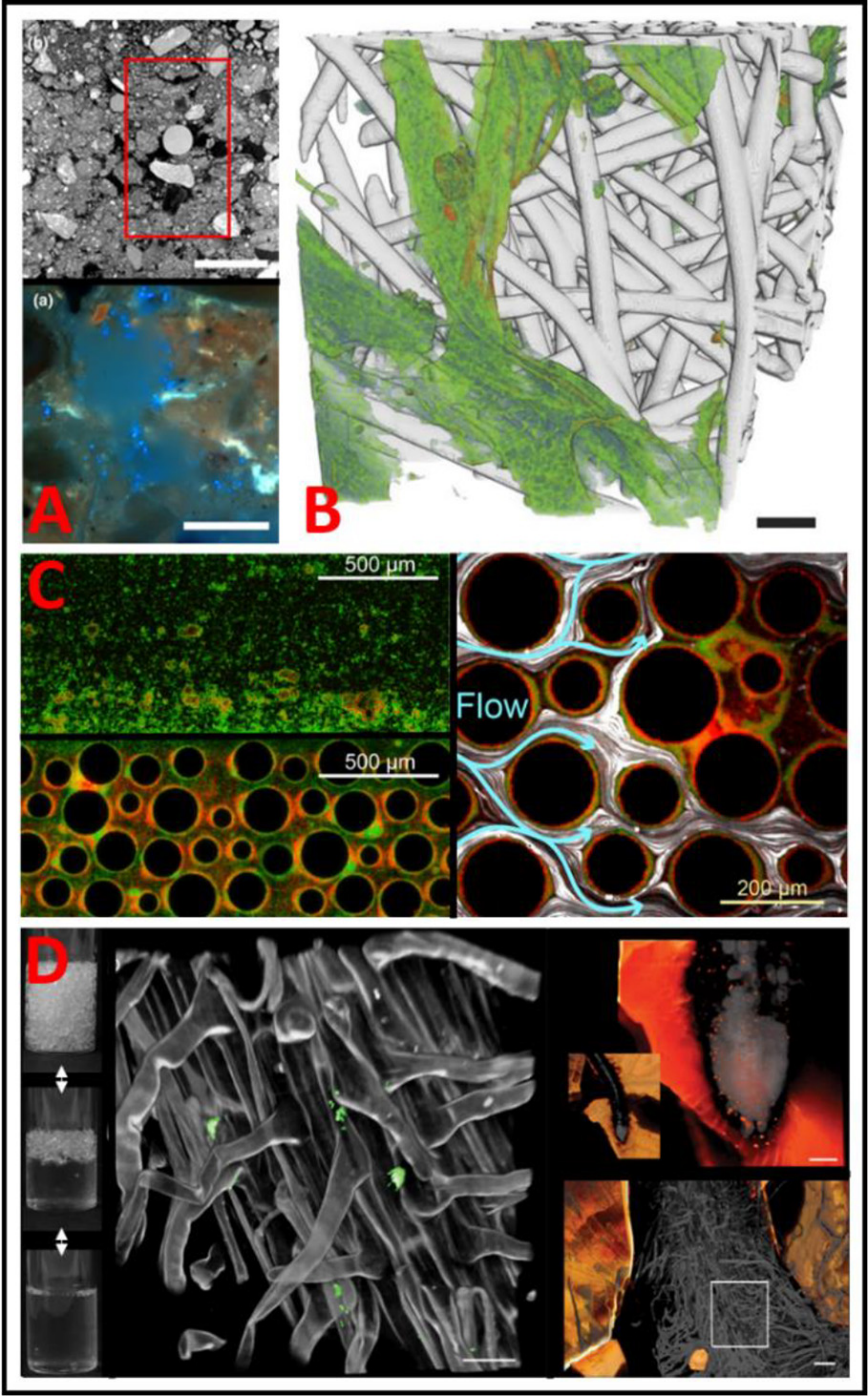
Sample images from methods developed for examining microbial interactions with physical environment. A- X-ray CT cross section of soil column with bacteria-inoculated glass bead (top panel, scale bar 5 mm) and fluorescence micrograph of cross-sectioned, resin impregnated soil column (bottom panel, scale bar 20 μm), showing DAPI stained (blue) *Pseudomonas fluorescens* in pore space. Adapted from Juyal et al. [26]. B- 3D rendering of nanoscale X-ray CT data showing fibroblast cells (green, nuclei red) within polymer scaffold (white) after 3 days of culture. Scale bar, 10 μm. Adapted from Bradley et al., [6]. C- Microfluidics chambers with either planar (top left) or obstacle (bottom-left and right) designs, showing two green- or red-fluorescence tagged strains of *Pseudomonas aeruginosa* and medium flow tracked with white fluorescent beads (right). Adapted from Nadell et al., [37]. D- Nafion pellets used as a substrate for plant growth, showing optical transparency when saturated with water (left). This transparent substrate allows roots (middle, scale bar 30 μm) to be imaged *in situ,* here with associated fluorescence-tagged *E. coli* (green). Top right, enlarged root with Nafion pellets in orange (scale bar 100 μm). Bottom right, nuclear RFP expression linked to auxin reporter (scale bar 54 μm). Adapted from Downie et al., [16]. (For interpretation of the references to colour in this figure legend, the reader is referred to the web version of this article.)

In a recent study, Juyal et al. [26] used the innovative approach of combining X-ray CT with soil thin-sectioning to examine the influence of soil structure on migration of *Pseudomonas fluorescens,* inoculated into soil columns filled with soil aggregates. After inoculating a glass bead with the bacterium and packing that within a soil column, the authors examined the three-dimensional structure of the surrounding soil pore-space by X-ray CT and characterised properties of the soil, such as the volume of pore space and proportion of pores connected to each other. Subsequent impregnation with resin and thin-sectioning enabled determination of the bacterial movement (and count) away from the inoculation point, by fluorescence stereomicroscopy (Fig. 2A). This revealed that the rate of spread of the bacteria was dependent on both the soil bulk density, pore connectivity, and interphase between solid surface and pore space, i.e., measurements only achievable at this scale with X-ray CT. This highlighted how the integration of several methods, also combined with the ability to place organisms at defined locations within a structure, could reveal previously unanswered questions in the field.

While there are many more examples of the use of X-ray CT for assessing soil structure [31], [29], [13], [52], [19], the applications to structured environments go beyond soils. Bradley et al. [6] used X-ray nanotomography – X-ray CT at sub-micron resolution – with Zernike phase-contrast (to enhance the subject/background contrast) for examining the distribution and depth of human fibroblast cells grown on polymer scaffolding. Importantly, this high-resolution X-ray CT could show the distribution of cells in relation to depth and pore size (Fig. 2B). This feature could be of considerable value for assessing microbe-environment interactions in future studies, albeit with the added challenge of typical microbial cells being smaller than human fibroblasts. Further examples of assessing biota-material interactions vary markedly in application, from concrete repairing properties of genetically modified bacterial strains [51], to the composition of corrosive by-products in ship wreckage materials, as many of these by-products are of bacterial origin [2]. Studies such as these highlight the multidisciplinary overlap between material science and microbiology.

#### 2.1.2 The use of X-ray CT data in conjunction with spatial simulation and modelling

The digital application of X-ray CT should also be considered in conjunction with computational modelling in that, because X-ray CT generates digitised three-dimensional structures, the data naturally lend themselves to computer simulation and modelling approaches. For example, there are several mathematical models describing theoretical growth patterns of microbial filaments within or on matrices, often demonstrating radial branching patterns, navigation within a two-dimensional environment or, in fewer cases, navigation in simulated three-dimensional spaces [5]. Models of growth and networks of fungal hyphae have been applied to the three-dimensional structures generated from X-ray CT data, including soil pore-architecture, to study microbial dynamics in soils with differing management histories. The fragmented pore space associated with a no-tillage agricultural site (where soil is not mechanically disturbed between crop rotations) was disadvantageous to fungal invasion, whereas the larger, more-connected pores of tilled soil were more prone to hyphal invasion [27]. In other examples, mathematical models applied to CT data enabled the modelling of fluid flow in porous structures such as marble to assess “microbial mortar” restoration [36].

#### 2.1.3 Drawbacks and other considerations in the use of X-ray CT

Thus far, the advantages of X-ray CT as a tool for characterising the structured environments of microorganisms has been emphasized, namely: the ability to characterise quantitatively and non-destructively certain spatial parameters of a sample (e.g. pore space) and, in some cases, the organisms within it; allowing for repeated measurements of the same sample over time; resolving structural detail that was previously difficult to obtain. However, X-ray CT is not without limitations for studies of this kind. A fundamental problem is that X-ray radiation is ionising and penetrative, meaning it can damage both structures and the microorganisms within them. In the polymer scaffold work discussed earlier, an exposure time of eight hours appeared to damage the polymer, creating large pores through the structure [6]. Care is also needed to ensure that total X-ray dose falls below the threshold that can damage the subject organism without compromising image quality and acquisition time [50], such as the ∼10 kGy (kGy) threshold reported to cause damage to soil-borne organisms [21]. This can limit study design as, even if not lethal, the X-ray dosages could cause mutagenesis with unpredictable consequences for microbial behaviour. Imaging resolution must also be considered, as sub-micron resolution scanning is currently limited to much smaller sample sizes than microtomography. For example, a detector with 1000–2000 pixels limits the sample size to ∼50 µm for a 50 nm resolution [48]. In contrast, imaging at lower resolutions risks missing features, such as pores, smaller than the scanning resolution. This should be borne in mind when interpreting data, for example by restricting conclusions to pores or other features of a size greater than the detection limit [26].

### 2.2 Fabrication (and application) of microfluidics devices and other structured environments

**Fig. 3:**
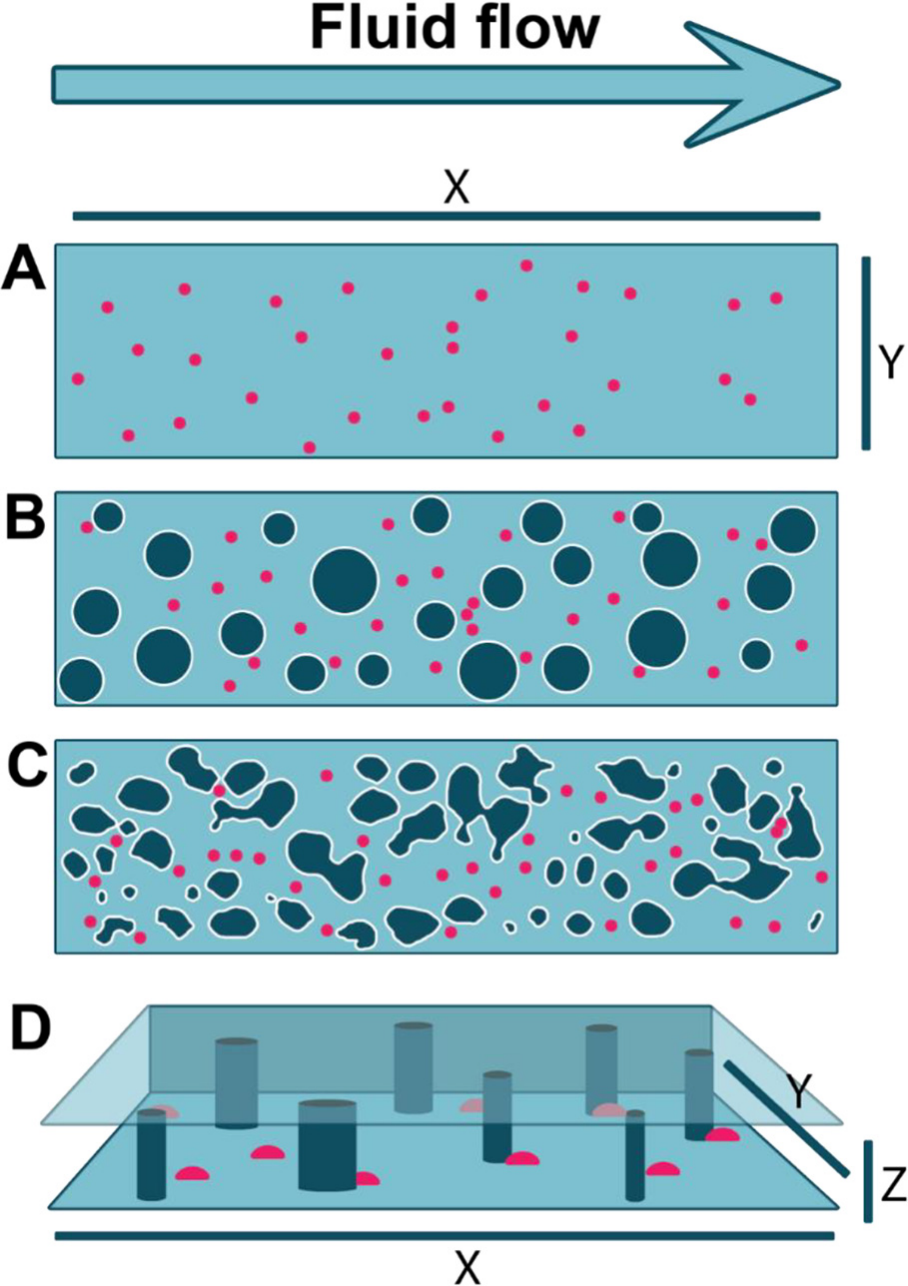
Schematic of potential microfluidic arrangements incorporating microorganisms (pink circles) and physical structures (dark green with white outline). A, B, C represent top-down views of devices, D shows side view of a chamber with similar arrangement to B. A- Planar microfluidics chamber with uniform fluid flow and microorganisms evenly exposed. - (For interpretation of the references to colour in this figure legend, the reader is referred to the web version of this article.)

Investigating microbial populations at the single cell and microcolony levels (as opposed to the bulk population) has gained significant interest in recent years [32], [23], [1]. However, this presents several challenges, including analysing and tracking organisms at the single-cell level, ideally in controlled (micro)environments. One approach to addressing these challenges is the use of microfluidic devices, here referring to devices which allow precise manipulation of fluids and fluid flow at the microlitre scale and below; typically achieved by introducing positive or negative pressure across confined channels. This precise manipulation of small liquid volumes allows fluid flow at defined rates, rapid transition between different fluids, and the ability to establish concentration gradients (e.g., nutrient, drug, etc) within a chamber. These devices have been used to study single cells in isolation or within a cell community and facilitate the measurement of a plethora of microbial phenotypes, e.g., cell morphology, division dynamics, protein expression, cell–cell interactions, gene regulation etc. [24], [9]. There is considerable value in using microfluidics to study controlled, homogeneous environments, such as by reducing the impact of environmental variation on microbial phenotypes of interest (Fig. 3A). However, structured environments can also be developed within microfluidics devices (Fig. 3B-D) to investigate the impacts of microscale structures on microbial phenotypes and processes [37], [25], [14].

#### 2.2.1 Microfluidics devices for investigating microbial biofilms and cell–cell interactions

Microbial biofilms were discussed earlier as structured environments in terms of the micro-topology concerned with biofilm adherence to surfaces. Moreover, biofilms also have complex internal structure, determined primarily by the spatial distributions of component organisms and their activities. The latter includes secretion of products, such as extracellular polymeric substances (EPS) that can facilitate microbial persistence by providing physical compartmentalisation and protection from external stressors [15]. Microfluidics has been used to establish biofilms and monitor growth and inherent metabolic dynamics under controlled conditions. In a recent study, *E. coli* monolayers were cultured in a device designed to establish a glucose gradient in a series of dead-end chambers containing the bacteria (measuring 40 µm × 60 µm) after introducing flow perpendicular to the chamber opening [13]. Fluorescence analysis of gene reporter expression indicated that cells at the front of the chamber metabolised glucose and secreted acetate whereas cells towards the back of the chamber (which were glucose limited) could metabolise the secreted acetate. Similar uses of dead-end microfluidics chambers have been employed to understand metabolic heterogeneity across yeast monolayers over time [34]. In these examples, the design of the microfluidic chambers produced the monolayer cell growth (a low ceiling height prevented cells from stacking vertically) and the apparent spatial division of metabolic specialisation, whereas the control of fluid flow facilitated nutrient switches without the perturbation of cells. A further methodological example of cell–cell interaction in structured environments is the use of several large chambers connected in parallel by narrow channels to generate fragmented structured environments, which have been shown to influence bacterial predator–prey dynamics [25].

The use of microfluidics also allows the study of how biofilm physical structure may influence competition between neighbouring organisms. For example, Nadell et al. [37] produced microfluidic devices with either no obstacles (planar) or with cylindrical columns of various diameters (producing disrupted flow) within the growth chambers (Fig. 2C). They introduced nutrient flow to compare biofilm formation in these structured environments. It was found that in the disrupted-flow model, a biofilm-defective mutant was lost in the flow-through at a slower rate when it was co-cultured with the wild-type. This was because wild-type biofilm formation could further disrupt flow and trap mutant cells. This example illustrates the new insights attainable by introducing physical structures within microfluidic chambers, as the interaction between the mixed genotypes was only evident in the structured chambers. In another example, microbial migration through a similar microfluidics structure was used to help demonstrate that motile *Pseudomonas putidi* cells could disperse more efficiently than non-motile mutants and were better able to navigate against the direction of flow across different scales [43]. This allowed the motile cells to explore more pore space relative to non-motile cells

Soil micromodels (i.e. 2D or 3D models of soil structure within a microfluidic system) provide excellent examples of integration with real-world structures [12], [42], [14]. Unlike the previous example, the structures within soil micromodels are created from computational simulations of soils (e.g. sandy loam soils), subsequently modified to remove closed pores so facilitating fluid flow through the entire model. Soil micromodels have been used to indicate impacts of microbial EPS on soil drying rates, which were on average ∼1.9 times slower in the presence of EPS-secreting compared with non-secreting strains of the bacterium *Sinorhizobium meliloti* [14]. These models have also been used to demonstrate the importance of protist behaviour on particle migration by tracking the movement of fluorescent beads within models over time in the presence or absence of the soil protozoa (*Colpoda* spp.) [42]*.* In principle, any scalable structure could be introduced into a microfluidics device, although there are potential fabrication complications with some structures, discussed below.

#### 2.2.2 Drawbacks of microfluidics

There are many important advantages of microfluidic-based methods, including the precise control of environmental conditions, the ability to structure the environment and to track many cells over time. However, there are also some limitations to the manufacture of microfluidic devices containing models of structured environments. Structures within the devices are typically three-dimensional extrapolations from floor to chamber-ceiling of two-dimensional shapes. For example, the soil micromodels described above are designed from two-dimensional cross sections from a three-dimensional soil structure, which are then extruded to produce a three-dimensional shape. A limitation related to such pseudo three-dimensional structures concerns pore-connectivity and porosity, in that the porosity of a two-dimensional slice from a three-dimensional model, that maintains complete connectivity, is expected to be higher than the overall porosity of the three-dimensional model [14]. It is possible to manufacture truly three-dimensional microfluidics structures, but this is more technically challenging than the simpler structures [10].

#### 2.2.3 Other methods of fabricating structured environments

Microfluidics devices can be considered fabricated structures, but other manufacturing approaches can also be used to prepare reproducible, structured environments. Such approaches may use materials relevant to the organisms’ natural habitats or reproduce complex three-dimensional structures using additive manufacturing technology [39], [35]. These approaches, while challenging, offer the ability to investigate systems in ways not otherwise accessible and are discussed below.

Recently, a method was developed for producing artificial soil aggregates with defined microbial composition, by sequential vortexing of ground soil together with microbial (yeast) inocula in layers [19]. This enabled a subsequent demonstration of stressor- and time-dependent protection of the microbial inoculum from environmental stress arising from encapsulation within a soil aggregate. These “manufactured” aggregates had similar structural characteristics as their natural counterparts, so providing realistic microorganism-bearing soil aggregates for laboratory studies. A key advantage over retrospective examination of natural soil structures is the ability to maintain the structured environment in defined conditions while manipulating the complexity of the system, such as the abundance and location of the microbial community.

There are of course advantages of using synthetic materials to mimic soil structures for microbiological studies. Ground pellets of Nafion (a sulfonated tetrafluoroethylene polymer) have been used as a substrate for cultivating plants and imaging root-microbe and root-nematode interactions [40], [17], [16]. Because these pellets have a refractive index similar to water, Nafion “soil” columns become optically transparent when saturated with water, allowing brightfield and fluorescence imaging of the plant roots and associated organisms in the structured matrix. Although this material lacks the chemical complexity of soils, it can be used to produce structures of defined particle-size distributions and, most importantly, to allow observation over time of root-biota interactions *in situ* at micron scale resolutions (Fig. 2D). Lysogeny Broth (LB) infused hydrogel particles have also been used in tracking bacterial migration through porous media [3]. These hydrogel particles were optically transparent and more uniform in diameter and sphericity than ground Nafion, enabling more robust control of the porosity of the medium. The study provided principles for predicting cellular migration over long time scales and distances.

Environments suitable for the study of microbial adhesion and biofilm growth have also been manufactured in the form of scratches and topologies in hygienic materials, produced by laser interference patterning at the nano to microscale [20] or mechanical scratching with high-resolution scratch testers at the microscale [47]. The fabrication of structures with a range of topologies allows greater understanding of the environmental or other factors that affect microbial surface-adhesion. For example, Helbig et al. [20] could propose that structures in which the cell:substrate contact area is similar to the cell size range yield the highest cell retention rates.

Additive manufacturing also provides an avenue for generating structured environments with high precision. Additive manufacturing is the process of printing successive layers of material on top of each other, although the methods of achieving this vary (see Ngo et al. [39] for an overview). Soil structural information acquired by X-ray CT has been subsequently used for printing three-dimensional soil-mimetic structures, in either Nylon 12 or resin with paraffin wax to preserve pore structure [30], [41]. Hyphal growth of fungi within the pores of the printed structures has also been demonstrated, showing that the structures support microbial growth and exploration [41]. In principle, a wide range of structured environments could be additively manufactured, provided that the material used for printing is biocompatible and that the structure can be computationally modelled.

When fabricating structures, some general precautions and limitations should be considered. For example, the fabrication material must be biocompatible, or at least non-toxic, and the optical or absorbance properties of the material should also be considered if the aim is to image or X-ray the structure and microorganisms. Care should also be taken when designing structures to ensure they are an appropriate proxy for the environment of interest (e.g. design of microfluidics chambers based on real soil geometry; [42], and this should be experimentally validated where possible (e.g. comparison of manufactured and natural soil structures by X-ray CT; [19]).

## 3 Summary and outlook

In this review, we highlight recent, novel and often multidisciplinary, methods for dissecting and examining the interplay between structured environments and microbial life. We emphasise the use of X-ray CT to characterise structures non-destructively and how this technology can be used in conjunction with other techniques to add further layers to analysis. Microfluidic devices and additive manufacturing can be used to reproduce structured environments, such as natural soil structures, and also create novel structures to examine how microorganisms may adapt to alternative physical forms. In addition, several standalone methods demonstrate further possibilities and applications for incorporating structure into microbial experimental systems. Taken together, these approaches and the insights they offer demonstrate the opportunities now available in this field.

While some of the studies highlighted here employed newly developed methods, modified or combined previously published approaches to examine a question in a way not achievable previously. It is likely that further cross-disciplinary integration of methods in this way will provide new future opportunities for peeling back the layers of complexity. For example, the ability to manufacture soil aggregates [19] in conjunction with analysis by DNA metabarcoding could enable tracking of microbial evolution within soil aggregates. Such advances help bring us closer to characterising and understanding the behaviour of microorganisms as it plays out in their natural, structured environments. This may in turn lead to a wider extrapolation of laboratory results to real-world settings, such as for predicting impacts on microbial communities of changes in environmental structure (e.g. arising from altered land use in the example of soil), or for improving efficacy of drug delivery in combatting biofilm mediated disease.

## CRediT authorship contribution statement

**Harry J. Harvey:** Conceptualization, Writing - original draft. **Ricky D. Wildman:** Writing - review & editing, Supervision. **Sacha J. Mooney:** Writing - review & editing, Supervision, Funding acquisition. **Simon V. Avery:** Conceptualization, Writing - review & editing, Supervision, Funding acquisition.
